# 4′-Methoxyresveratrol Alleviated AGE-Induced Inflammation via RAGE-Mediated NF-κB and NLRP3 Inflammasome Pathway

**DOI:** 10.3390/molecules23061447

**Published:** 2018-06-14

**Authors:** Wenzhe Yu, Mengru Tao, Yueliang Zhao, Xiaoqian Hu, Mingfu Wang

**Affiliations:** 1 College of Food Science and Technology, Shanghai Ocean University, No. 999 Hu Cheng Huan Road, LinGang New City, Shanghai 201306, China; wzyu0129@outlook.com (W.Y.); tmemory139@163.com (M.T.); ylzhao@shou.edu.cn (Y.Z.); 2 Shanghai Engineering Research Center of Aquatic-Product Processing & Preservation, Shanghai 201306, China; 3 School of Biological Sciences, The University of Hong Kong, Pokfulam Road, Hong Kong, China

**Keywords:** 4′-methoxyresveratrol, MAPK, oxidative stress, RAGE, NF-κB, NLRP3 inflammasome

## Abstract

Advanced glycation end products (AGEs) could interact with the receptor for AGE (RAGE) as a sterile danger signal to induce inflammation. 4′-methoxyresveratrol (4′MR), a polyphenol derived from Dipterocarpaceae, has not been studied for its anti-inflammation effects. In the present study, we sought to explore the protective role of 4′MR in AGEs-induced inflammatory model using RAW264.7 macrophages. 4′MR significantly inhibited gene expression of pro-inflammatory cytokines and chemokines, such as interleukin 1β (IL-1β), interleukin 6 (IL-6), tumor necrosis factor-alpha (TNF-α) and monocyte chemoattractant protein-1 (MCP-1), as well as two typical pro-inflammatory enzymes, inducible nitric oxide synthase (iNOS) and cyclooxygenase 2 (COX2). Besides, 4′MR significantly decreased oxidative stress, demonstrated by levels of ROS production, protein carbonyl and advanced oxidation protein product via down-regulation of NADPH oxidase. Further analysis showed that 4′MR attenuated the RAGE overexpression induced by MGO-BSA. It also blocked the downstream signal of AGE-RAGE, particularly, MAPKs including p38 and JNK, and subsequently reduced NF-κB activation. Additionally, 4′MR significantly abated the activation of NOD-like receptor pyrin domain containing 3 (NLRP3) inflammasome including NLRP3 and cleaved caspase-1 and reduced the secretion of mature IL-1β. Taken together, our results suggest that the anti-inflammatory effect of 4′MR is mainly through suppressing RAGE-mediated MAPK/NF-κB signaling pathway and NLRP3 inflammasome activation. 4′MR could be a novel therapeutic agent for inflammation-related diseases.

## 1. Introduction

AGEs are a group of heterogeneous compounds that have been implicated in the progression of different diseases such as arthritis, Alzheimer’s disease and diabetes, and are also critical for the propagation of inflammatory responses [1]. The formation of AGEs occurs not only under hyperglycemia and hyperlipidemia condition, but also under other homeostatic imbalances such as aging [2], redox imbalance [3] or autoimmune disease [4]. As a sterile danger signal, AGEs can interact with cell surface receptors including RAGE, resulting in activation of certain signaling pathways and overproduction of cytokines, chemokines and other inflammatory mediators that eventually induce inflammation [5]. The activated macrophages have been implicated in the progression of some metabolic and inflammatory diseases including obesity, diabetes and atherosclerosis [6]. However, the cellular mechanism behind AGEs-mediated pathogenic disorders in macrophage needs to be further investigated. 

Nuclear factor kappa B (NF-κB) is a group of structurally related transcription factors that are essentially found in all cell types [7]. The exposure of cells to a variety of extracellular stimuli leads to the rapid phosphorylation of NF-κB, and ultimately regulates gene transcription. To date, AGEs as a group of exogenous substances involved in NF-κB activation, which is responsible for the maintenance and amplification of the signal with a subsequent induction of the inflammatory response [8].

In recent years, the nucleotide-binding oligomerization domain (NOD)-like receptor (NLR) pyrin domain containing 3 (NLRP3) is discussed extensively as a novel cell stress signal [9]. A growing number of studies have provided solid evidence that NLRP3 inflammasome can be activated by AGEs [10,11]. Furthermore IL-1β, an inflammatory cytokine especially regulated by NLRP3 inflammasome, plays an essential role in the regulation of numerous autoinflammatory or autoimmune pathologies [9]. Even though the exact mechanism behind the activation of NF-κB and NLRP3 by AGEs remains to be fully elucidated, the sensing of cellular stress signals, especially over-production of reactive oxygen species (ROS) or overexpression of RAGE links to a direct activation of these inflammatory processes. 

Polyphenols are one group of important secondary metabolites in plants, attracting more and more attentions for their health effects, which show various biological activities, such as acting as potent antioxidants and free radical-scavengers [12]. Moreover, they have been proven to protect from neurodegenerative diseases, insulin resistance, and especially inflammation-related diseases [13]. 4′-Methoxyresveratrol (4′MR) (3,5-dihydroxy-4′-methoxystilbene) is a plant stilbene found in the Dipterocarpaceae and Gnetaceae with little information available for its bioactivity. It has been reported that 4′MR shows antiandrogenic activity in prostate cancer cells [14] and displays antifungal activity in vitro [15]. However, there is no report on 4′MR targeting inflammation in any model. In this study, we evaluated whether 4′MR has protective effects on AGE-induced inflammation and the involvement of NF-κB and NLRP3 inflammasome in RAW264.7 macrophages.

## 2. Results

### 2.1. 4′-Methoxyresveratrol Modulated Pro-Inflammatory Markers Expression Induced by AGEs

**Figure 1:**
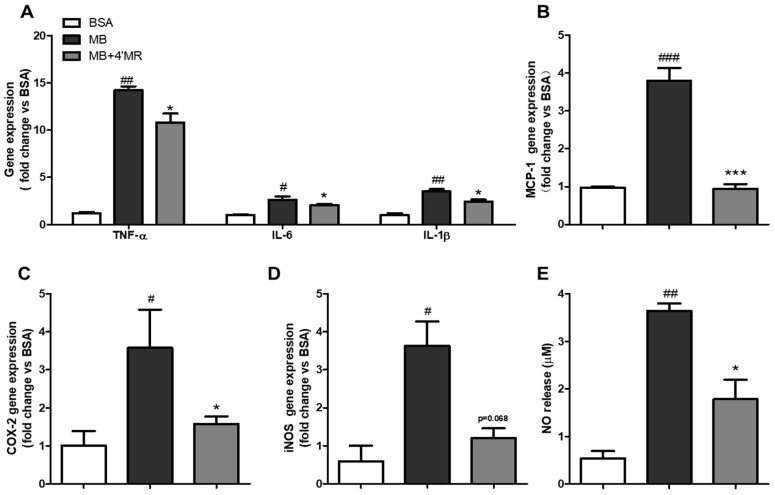
4′-Methoxyresveratrol modulated pro-inflammatory gene expression induced by MB in RAW264.7 macrophages. (**A**–**D**) TNF-α, IL-6, IL-1β, MCP-1, COX2, and iNOS mRNA levels were determined by qPCR. Gene expression was normalized to 18S. (**E**) NO level was analyzed by Griess method in culture supernatant. Cells were treated with 200 μg/mL of BSA or MB with or without 10 μM 4′MR for 24 h. All data are averages of triplicates from three separate experiments. # *p* < 0.05, ## *p* < 0.01, ### *p* < 0.001 vs. BSA; * *p* < 0.05, *** *p* < 0.001 vs. MB. 4′MR, 4′-Methoxyresveratrol.

To determine the influence of 4′-methoxyresveratrol on the pro-inflammatory genes expression in AGEs-induced cells, levels of IL-6, TNF-α, IL-1β, MCP-1, iNOS and COX-2 and in cells were measured by qPCR. As shown in Figure 1A–D, compared with the BSA-treated group, the mRNA expression of all detected genes in MGO-BSA (MB)-treated group (200 µg/mL) were remarkable upregulated at 24 h treatment. In contrast, the levels of those mRNA showed downregulation at different degrees in 4′MR-treated group (10 µM).

The enhanced expression of inducible nitric oxide synthase (iNOS) was the main reason for NO production which was believed to be widely involved in the development of inflammation [16]. Griess method result (Figure 1E) showed the NO level was also increased in AGEs-treated group compared with the BSA-treated group (*p* < 0.01), while the co-treatment with 4′MR decreased 2.04-folds the NO level in the supernatant at 24 h treatment (*p* < 0.05).

### 2.2. 4′-Methoxyresveratrol Alleviated Oxidative Stress Induced by AGEs

**Figure 2:**
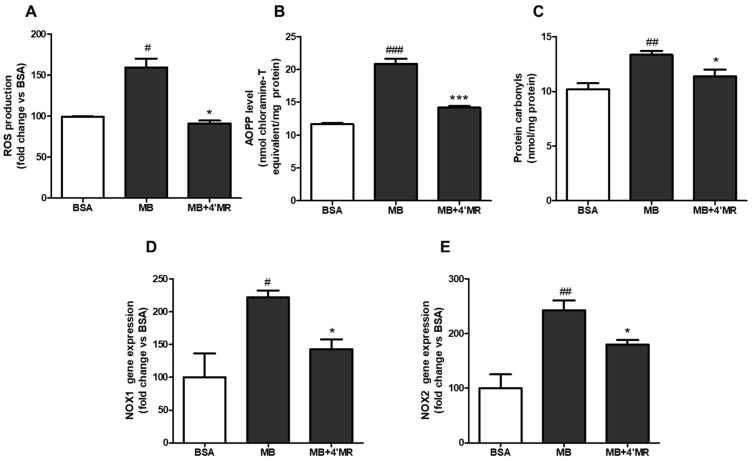
4′-Methoxyresveratrol alleviated oxidative stress induced by MB in RAW264.7 macrophages. (**A**) The ROS production induced by MB was determined by DCFH-DA. (**B**,**C**) The levels of protein carbonyls (**B**) and AOPP (**C**) were measured by a protein carbonyl assay kit and chemical method, respectively. (**D**,**E**) mRNA expressions of NOX1 (**D**) and NOX2 (**E**) were measured by qPCR. Gene expression was normalized to 18S. Cells were treated with 200 μg/mL BSA or MB with or without 10 μM 4′MR for 24 h. All data are averages of triplicates from three separate experiments. # *p* < 0.05, ## *p* < 0.01, ### *p* < 0.001 vs. BSA; * *p* < 0.05, *** *p* < 0.001 vs. MB. 4′MR, 4′-Methoxyresveratrol.

Oxidative stress was considered to be caused by imbalance between oxidant and antioxidant system when cells or tissues responded to excess xenobiotics or bacterial invasion [17]. To understand whether 4′MR could alleviate AGEs-induced oxidative stress, we first examined its ability to regulate reactive oxygen species (ROS) production and the formation of oxidative protein products. As shown in Figure 2A–C, the contents of ROS and oxidative protein products were significantly increased in MB-treated group compared with BSA-treated group (200 µg/mL) after 24 h treatment, while cells co-treated with 10 µM 4′MR could reverse the action of AGEs. The level of ROS, advanced oxidation protein product (AOPP), and protein carbonyls were reduced approximately 43% (*p* < 0.05), 32% (*p* < 0.001) and 15% (*p* < 0.05), respectively.

To figure out how 4′-MR could inhibit the progression of oxidative stress, the expressions of NADPH oxidase (NOX) mRNA were measured by qPCR. As shown in Figure 2D,E, the mRNA levels of NOX1 and NOX2 were significantly higher in MB-treated cells than in BSA-treated cells (200 µg/mL) after 24 h treatment, whereas these effects were attenuated approximately 35.4% (*p* < 0.05) and 25.8% (*p* < 0.05), respectively, by co-treatment with 10 µM of 4′MR.

### 2.3. 4′-Methoxyresveratrol Suppressed mRNA and Protein Level of RAGE

**Figure 3:**
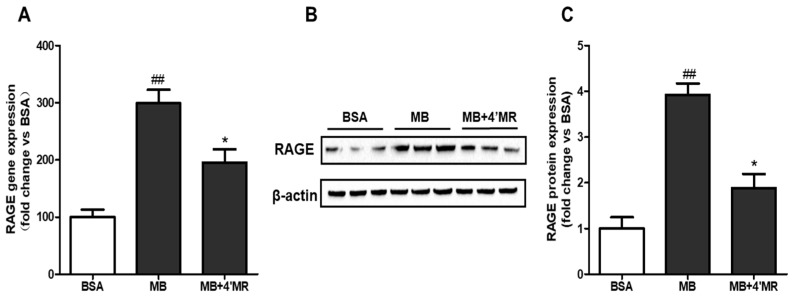
4′-Methoxyresveratrol inhibited RAGE expression induced by MB in RAW264.7 macrophages: (**A**) RAGE mRNA level was determined by qPCR. Gene expression was normalized to 18S; (**B**) representative Western blotting images of total RAGE protein expression; and (**C**) quantification of fold-change of RAGE protein expression. Cells were treated with 200 μg/mL BSA or MB with or without 10 μM 4′MR for 24 h. All data are averages of triplicates from three separate experiments. ## *p* < 0.01 vs. BSA; * *p* < 0.05 vs. MB. 4′MR, 4′-Methoxyresveratrol.

Receptor-dependent changes, such as the binding of AGEs to the cell surface RAGE, have an essential role in chronic inflammatory diseases. We then measured RAGE in macrophage using qPCR and Western blot, respectively. In MB-treated group (200 µg/mL), the levels of RAGE mRNA and protein were significantly increased compared to BSA-treated group (*p* < 0.01) and these effects were inhibited in 4′MR-treated group (*p* < 0.05) at 24 h treatment (Figure 3). Our data indicated that 10 µM of 4′MR had a potent effect in signal transduction which is bound up with RAGE.

### 2.4. 4′-Methoxyresveratrol Modified MAPKs/NF-κB Pathway Induced by AGEs

**Figure 4:**
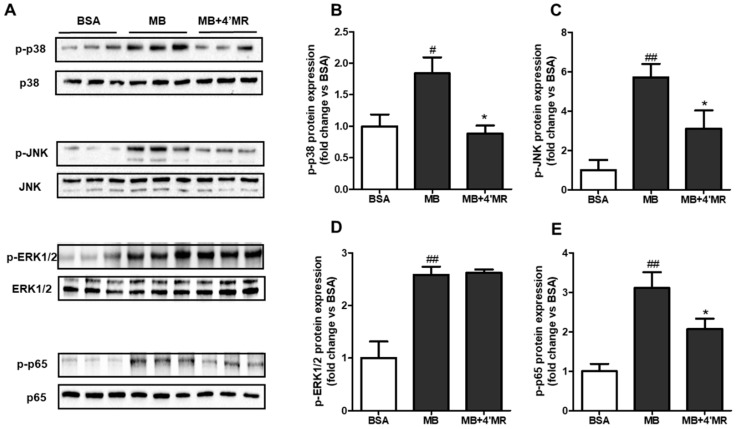
4′-Methoxyresveratrol down-regulated MAPKs/NF-κB signaling pathway activation induced by MB in RAW264.7 macrophages. Cells were starved for 4 h and then treated with 200 µg/mL BSA or MB with or without 10 µM 4′MR for 45 min. (**A**) Representative Western blotting images of phosphorylated and total p38 MAPK, JNK, ERK1/2 and p65. (**B**–**E**) Quantification of fold-change of: phosphorylated p38 MAPK (**B**); JNK (**C**); ERK1/2 (**D**); and p65 (**E**). All data are averages of triplicates from two separate experiments and shown as mean ± SD. # *p* < 0.05, ## *p* < 0.01 vs. BSA; * *p* < 0.05 vs. MB. 4′MR, 4′-Methoxyresveratrol.

AGEs binding to RAGE has been shown to activate multiple cellular signaling cascades [5]. To further elucidate the regulatory mechanism of 4′MR on cell disorders induced by AGEs, we analyzed the key regulators involved in NF-κB signaling. Here, the expression levels of p-p38 MAPK, p-ERK1/2, p-JNK, and p-p65 were measured by Western blot. As shown in Figure 4, 200 µg/mL MB treatment induced the activation of MAPKs/NF-κB pathway by enhancing the phosphorylation of p38 MAPK (*p* < 0.05), ERK1/2 (*p* < 0.01), JNK (*p* < 0.01), and p65 (*p* < 0.01) compared to the BSA-treated group at 45 min treatment. The results of co-treatment with 4′MR showed that the elevated protein expressions triggered by MB of p-p38 MAPK, p-JNK, and p-p65 were significantly inhibited. However, the elevated protein level of p-ERK1/2 induced by MB was not changed when cells were co-treated with 4′MR at 45 min, indicating that 4′MR could decrease the pro-inflammatory gene expression without the involvement of ERK1/2 pathway. 

### 2.5. 4′-Methoxyresveratrol Counteracted the Activation of NLRP3 Inflammasome Induced by AGEs

**Figure 5:**
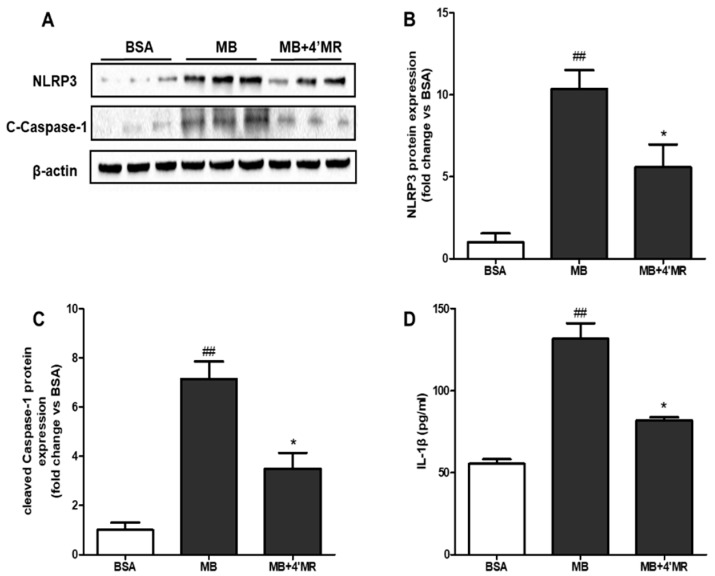
4′-Methoxyresveratrol counteracted NLRP3 inflammasome activation induced by MB in RAW264.7 macrophages. Cells were treated with 200 μg/mL BSA or MB with or without 10 μM 4′MR for 24 h. (**A**) Representative Western blotting images of total NLRP3 and cleaved caspase-1 protein expression; (**B**,**C**) Quantification of fold-change of NLRP3 (**B**) and cleaved caspase-1 (**C**) protein expression; and (**D**) IL-1β secretion was analyzed by ELISA in culture supernatant. All data are averages of triplicates from two separate experiments and shown as mean ± SD. ## *p* < 0.01 vs. BSA; * *p* < 0.05 vs. MB. 4′MR, 4′-Methoxyresveratrol.

It was reported that AGEs could lead to the activation of inflammasome via oxidative and inflammatory stress [9], so we further examined whether the activation of inflammasome could be suppressed by 4′MR treatment through measuring NLRP3 and bioactive cleaved caspase-1. As shown in Figure 5A–C, 200 µg/mL MB treatment triggered the first step of activating NLRP3 inflammasome, which enhanced the protein level of NLRP3 (*p* < 0.01), and activated the second step, leading to the increased protein level of cleaved caspase-1 (*p* < 0.01). Compared with the MB-treated group, the protein levels of NLRP3 and cleaved caspase-1 were reduced approximately 1.85 and 2.04 folds (*p* < 0.05), respectively, in 4′MR- treated group at 24 h. Furthermore, NLRP3 inflammasome activated by non-microbial danger signals could ultimately bring about the maturation and secretion of pro-inflammatory cytokines IL-1β [18]. Thus, we next investigated the specific secretion of IL-1β in culture medium by ELISA after cells being treated for 24 h. The treatment with MB significantly increased IL-1β secretion compared to BSA-treated macrophages, whereas this effect was attenuated by 4′MR co-treatment (Figure 5D).

## 3. Discussion

The accumulation of AGEs in cells and tissues has been found in healthy persons and persons with metabolic disorders [19]. Endogenous formation of AGEs is a complex process, and reactive carbonyl species such as GO and MGO are indispensable mediators which react with amino acids, proteins or lipids to form AGEs, and induce dysfunction in macrophages [20]. We previously reported an in vitro model stimulated by MGO-BSA showing oxidative stress and inflammation in RAW264.7 cells [21]. In this study, the same model was used. 

AGEs are known to disturb cell functions via interaction with a series of cell surface receptors [22]. The most studied one is RAGE, a member of the immunoglobulin superfamily. The expression of RAGE depends on the cell type and is regulated in response to changes of the extracellular environment [2,23]. We showed that both mRNA and protein expression of RAGE were significantly increased by AGE compared to control group, while the overexpression could be reversed by the co-treatment with 4′MR, a resveratrol-like stilbene. Furthermore, 4′MR showed a significant inhibitory effect on AGEs-induced oxidative stress and the expression of inflammatory biomarkers. These results indicated that 4′MR might be involved in RAGE receptor-ligand axis via inhibiting RAGE expression. Certain natural substances such as curcumin have been proven to own anti-inflammatory activity by suppressing AGE-RAGE associated vascular injury and long-term complications of diabetes [24]. Therefore, the inhibition of inflammatory processes induced by AGEs-RAGE axis was considered as a good potential intervention target for therapeutic purposes. To a certain extent, 4′-methoxyresveratrol attenuated the development of AGEs related chronic diseases in vitro.

Oxidative stress was a series of adaptive responses caused by the imbalance between ROS and the antioxidant system [25]. Several studies demonstrated that mild oxidative stress showed positive effect on metabolism due to the defense role of ROS in pathogens [26]. However, over-elevated ROS levels led to toxic effects, including protein oxidation, DNA damage, lipid peroxidation and activation of intracellular signaling such as MAPK, phosphoinositide 3-kinase (PI3K) and protein tyrosine phosphatases [17]. In the present study, 4′MR significantly inhibited the ROS production in AGEs-treated cells. In addition, 4′MR also reduced the content of protein carbonyls and AOPP which were typical biomarkers of oxidative stress when the production of ROS was excessive. The beneficial effect of 4′MR was similar with resveratrol, which was reported to attenuate RAGE/ROS pathway induced by AGEs in mouse macrophages [27]. Some studies suggested that methoxyl group on stilbene compounds might increase their bioavailability and antioxidant capacity compared to resveratrol in vitro and in vivo [28,29]. Thus, 4′MR might possess a greater ability to scavenge free radicals in biological systems, agreeing with our findings. The NOX family is a transmembrane protein transporting electrons across membranes. It is considered as the “professional” enzymatic source of cellular ROS, and therefore accepted to be a major contributor to cell dysfunction [30]. In our study, NOX1/2 showed a marked increase in AGEs-treated cells, and the co-treatment with 4′MR reduced their gene expression. A recent research reported that the abrogation of ROS production by a RAGE inhibitor was dependent on NOX2 in rat alveolar epithelial cells, indicating the role of RAGE in mediating ROS generation [31]. Our results demonstrated similar results that 4′MR inhibited ROS production partly via down-regulation of RAGE expression and then NOX expression. 

As a pattern recognition receptor, the overexpression of RAGE will trigger several signal transmission events, involving, at least in part, p21ras, MAPK and PI3K/Akt [32]. These signals eventually lead to NF-κB activation [5], which further mediates different cellular process including inflammation, apoptosis and proliferation [7]. NF-κB can be sequestered by the inhibitor of κB (IκB) combined with RelA (p65) and the latter can be released upon phosphorylation at Ser536 when activated. NF-κB is the primary signal transduction molecule activated by AGEs via MAPK signaling cascades, extensively involved in the inflammatory response [33]. As an example, Yeh et al. proved that p38, MAPK and JNK were required for NF-κB activation by AGE (CML adducts) by using relevant inhibitors in vitro [34]. To clarify the regulatory mechanism of 4′MR on cell disorders induced by AGEs, the key regulators involved in NF-κB signaling were measured. After treatment with AGEs, the phosphorylation of p38, ERK and JNK, as well as p65 were increased. These results were in agreement with our previous study that AGEs induced inflammation via MAPK and NF-κB pathways in RAW264.7 cells [21]. In the study, the co-treatment with 4′MR was found to significantly reduce the activation of NF-κB interacting with p38 and JNK, but not ERK. This phenomenon was partly in agreement with Chuang et al. who found that TNF-α mediated ERK1/2 activation in primary human adipocytes was not suppressed by the pretreatment with trans-resveratrol [35]. Furthermore, ROS was also necessary for sustaining phosphorylation of p38 [36] and JNK [37] through inhibiting the inactivating phosphatases, and the further NF-κB activation. As previously described, 4’MR treatment reduced AGEs-enhanced ROS production, indicating that the preventive role of 4′MR on inflammation was partly via ROS-reliant NF-κB. As the master transcriptional regulator of the inflammation process, NF-κB was able to induce the expression of a variety of pro-inflammatory genes, thereby ultimately augmented their synthesis and secretion [33]. Our data showed that AGEs increased the mRNA expression of MCP-1, IL-6, TNF-α, IL-1β, COX-2 and iNOS, while these inflammatory genes were inhibited more or less by the co-treatment with 4′MR. It could be speculated that 4′MR attenuated inflammatory gene expression may be through inhibiting NF-κB activation. 

Recently, NLRP3 inflammasome has been extensively studied as an unexpected sensor for the metabolic stress and inflammation to induce the maturation of IL-1β [9]. NLRP3 inflammasome is a cytosolic macromolecular complex composed of NLRP3 receptor, apoptosis-associated speck-like protein containing (ASC), caspase-1 and/or caspase-11 [9]. The canonical activation of NLRP3 inflammasome requires an initial signal regulated by NF-κB for transcription of NLRP3 and pro-IL-1β, followed by a second signal that assembles the NLRP3 inflammasome which contributes to the caspases-1 cleavage and further IL-1β maturation and secretion [18]. Song et al. recently demonstrated that RAGE/NF-κB pathway could activate NLRP3 inflammasome and cause IL-1β maturation in nucleus pulposus cells [26]. As 4′MR could block RAGE/NF-κB pathway, it was very likely that 4′MR would also suppress NLRP3 inflammasome activity as the downstream signal of NF-κB. Just as we proposed, the stimulated protein expression of NLRP3, cleaved caspases-1 and IL-1β secretion caused by AGEs, were dramatically reduced with the treatment of 4′MR in RAW264.7 cells. The reduced IL-1β secretion might result from a combined inhibition of both IL-1β mRNA expression and caspases-1 cleavage activity. Moreover, growing evidence indicates a crosstalk between the ROS and NLRP3 inflammasome pathway [9]. Scavengers of ROS, inhibitors or knockdown of NOXs are found to suppress NLRP3 activation in response to stimulants such as crystals, LPS [18] and AGEs [10]. Therefore, the attenuated activation of NLRP3 inflammasome by 4′MR might also be related with reduced ROS. The results are consistent with the study of Cai et al. who reported that procyanidins alleviated morphine tolerance via diminishing ROS and suppressing activation of NLRP3, MAPKs and NF-κB pathways in microglia [38]. In addition, Deng et al. showed that endothelial dysfunction and inflammation induced by AGEs could be alleviated by irisin via blocking ROS/NLRP3 inflammasome pathway [10]. As NLRP3 inflammasome definitely participated in host defense and auto-inflammatory disorders, the discovery of its potential inhibitors would be extremely useful for remission of some inflammatory diseases. Taken together, the novel protective effect of 4′MR was reported for the first time, suggesting that it would be a promising candidate for treatment of disorders induced by AGEs. 

**Figure 6:**
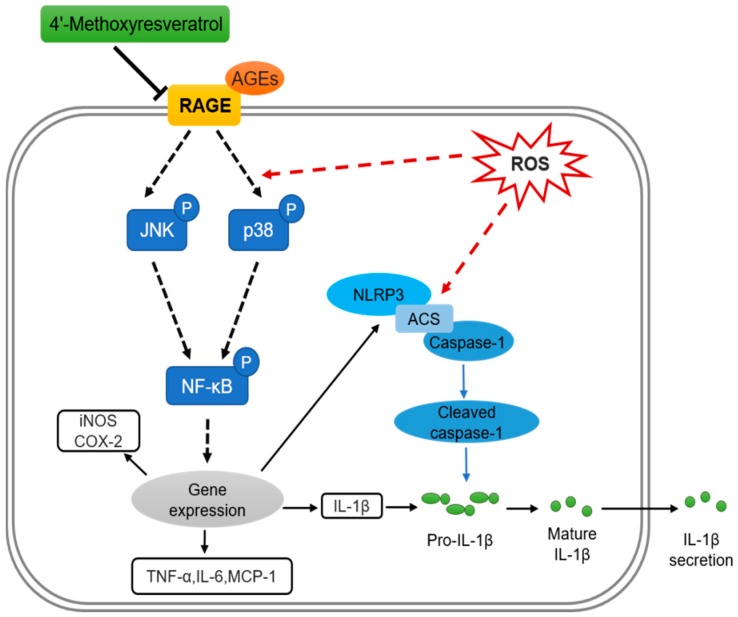
Schematic representation of protective effects of 4′-Methoxyresveratrol against MB-induced cell disorders in RAW264.7 cells.

In summary, the present data indicate the putative mechanism that 4′MR exerted a beneficial inhibition on pro-inflammatory gene expression and IL-1β secretion in an AGEs-induced cell model. The anti-inflammatory effect of 4′MR was mainly through blocking RAGE-mediated signaling pathway interacting with MAPKs and NF-κB, as well as NLRP3 inflammasome activation (Figure 6). Considering the worldwide rapid emergence in metabolic diseases due to the increased AGEs formation, 4′MR could be a therapeutic strategy for suppressing these diseases induced by AGEs.

## 4. Materials and Methods

### 4.1. Materials and Reagents

Dulbecco’s modified Eagle medium and penicillin–streptomycin solution were purchased from GIBCO (Grand Island, NY, USA). Fetal bovine serum was provided by Hyclone (Logan, UT, USA). Monoclonal rabbit antibodies, including anti-p38 MAPK, anti-phospho-p38 MAPK, anti-ERK1/2, anti-phospho-ERK1/2, anti-JNK, anti-phospho-JNK, anti-p65, anti-phospho-p65, NLRP3, and cleaved caspase-1 were obtained from Cell Signaling Technology (Boston, MA, USA). Receptor for AGE (RAGE) monoclonal rabbit antibody was purchased from AbCam (Cambridge, MA, USA). HRP-marked anti-β-actin antibody was supplied by Biorad (San Diego, CA, USA). 4′-Methoxyresveratrol (3,5-dyhydroxy-4′-methoxylstilbene) was purchased from Great Forest Biomedical (Hangzhou, China). BSA was obtained from ABCONE (Shanghai, China). KI and acetic acid were obtained from Sangon Biotech (Shanghai, China). Methylglyoxal, 2’,7’-dichlorodihydrofluorescein diacetate and other reagents were purchased from Sigma (St. Louis, MO, USA).

### 4.2. Cell Culture

Mouse RAW264.7 macrophages were purchased from Shanghai Institute of Cell Biology (Shanghai, China). Cells were maintained in 5% CO_2_ atmosphere at 37 °C in Dulbecco’s modified Eagle medium which was supplemented with 10% (*v*/*v*) fetal bovine serum plus 1% (*v*/*v*) penicillin–streptomycin solution (DF10). There was no significant cytotoxic effect of 4′MR on RAW264.7 macrophages under 30 µM, so the 10 µM 4′MR was used in this study (data not shown).

### 4.3. Preparation of Methylglyoxal-Modified AGEs

Glycation of bovine serum albumin (BSA) mediated by methylglyoxal (MGO), as the source of AGEs, was prepared according to the procedure published by our pervious study [21]. In brief, the final concentration of 10 mg/mL endotoxin-free BSA dissolved in phosphate-buffered saline (PBS) was incubated with or without 55 mM MGO at 37 °C for six days. The final MGO-BSA (MB) was stored at −20 °C until use after dialyzing for two days against PBS at 4 °C for removing non-reacted MGO and filtrating using 0.22 µm filter membrane. To ensure the significance of this study, the fluorescence intensity of MB was 70-fold higher than that of BSA measured with emission at 440 and excitation at 370 nm by a microplate reader. The MGO-BSA content was expressed as the concentration of non-glycated BSA.

### 4.4. Intracellular ROS Production Measurement

The effect of 4′-methoxyresveratrol on ROS production was determined by a method according to our previous study [21]. For quantification of intracellular ROS level, RAW264.7 macrophages were plated in a 96-well plate for 24 h, and then further stimulated with 200 µg/mL BSA or MB with or without 10 µM of 4′-methoxyresveratrol for 24 h. After the cultivation, supernatant was aspirated and cells were washed by warm PBS twice. Then cells were incubated with 25 µM 2’,7’-dichlorodihydrofluorescein diacetate (DCFH-DA) in DF10 for 1 h at 37 °C. After that, cells were washed with warm PBS twice. Finally, the fluorescence intensity of DCFH-DA-activated cells was determined by emission at 485 nm and excitation at 528 nm employing a microplate reader after adding 200 µL HBSS to each well. Results were expressed as a percentage of non-glycated BSA.

### 4.5. Protein Carbonyl and AOPP Levels Measurement

After treatment with 200 µg/mL BSA or MB with or without 10 µM 4′-methoxyresveratrol for 24 h, lysates from the cell culture were prepared using a homogenizer (ULTRA-TURRAX, IKA, Staufen, Germany). The concentration of total protein was measured by a BCA protein assay kit. Protein carbonyl expressed as nmol per mg total protein measured by a Protein Carbonyl Assay Kit according to the manufacturer’s protocol (Nanjing Jiancheng Bioengineering Institute, Nanjing, China). The content of advanced oxidation protein products (AOPP) was determined as described by our previous study [21]. In brief, the standard curve was draw by 0, 10, 20, 40, 80, and 100 µmol/L chloramine-T and 300 µL of protein lysates were used for sample detection. Seventy-five microliters of 1.16 M KI and 150 µL of acetic acid were added into the tubes which served as reactants with chloramine-T or samples. After that, their absorbance was measured by microplate reader at 340 nm immediately. The AOPP quantity was calculated with respect to chloramine-T and results showed in nmol of chloramine-T equivalent per mg total protein.

### 4.6. RNA Isolation and qPCR Analysis

RAW264.7 macrophages (10^6^ cells/well) were cultured in DF10 supplemented with 200 µg/mL BSA or MB with or without 10 µM 4′-methoxyresveratrol for 24 h, the total RNAs were extracted with a standard TRIzol (Life Technologies, Foster City, CA, USA) method according to the manufacturer’s instructions. The concentrations of extracted RNAs were measured by a spectrophotometer (Nanodrop, Invitrogen, San Diego, CA, USA). The cDNA synthesis was performed with reverse transcription by a PrimeScript RT reagent Kit (Takara, Shanghai, China) according to the manufacturer’s instructions. A real-time PCR quantitation of target genes was performed using QuantiFast SYBR-Green RT-PCR kits (Roche, Basel, Switzerland). 18S expression was used as an internal control. The sequences of forward and revere primers used are shown in Appendix A (Table A1). All the primers were synthetized by Sangon Biotech (Shanghai, China). The measurements were analyzed using the ΔCT method with LightCycler 96 (Roche, Basel, Switzerland) in the qPCR apparatus. 

### 4.7. Measurement of NO and IL-1β Levels

RAW264.7 macrophages (10^6^ cells/well) were cultured in DF10 supplemented with 200 µg/mL BSA or MB with or without 10 µM 4′-methoxyresveratrol for 24 h, and supernatants were collected for nitric oxide (NO) and interleukin 1β (IL-1β) measurement. Levels of NO and IL-1β were determined by a mouse NO and IL-1β ELISA kit (R&D systems, Inc., Minneapolis, MN, USA) according to the manufacturer’s instructions.

### 4.8. Protein Extraction and Western Blot Analysis

RAW264.7 macrophages (10^6^ cells/well) were cultured in DF10 supplemented with 200 µg/mL BSA or MB with or without 10 µM 4′-methoxyresveratrol for 45 min after being starved for 4 h in six-well dishes. Then, cells were washed with pre-cooled PBS twice and lysed with 200 µL of RIPA lysis buffer (Sangon Biotech, Shanghai, China) on ice for 15 min. The lysates were subsequently transferred into 2 mL tubes for collecting supernatant by centrifugation (12,000× *g*, 10 min, 4 °C). The concentration of protein was determined by a BCA protein assay kit. A total of 30 µg protein was run per lane on SDS-PAGE (Biorad, San Diego, CA, USA) and transferred onto PVDF membranes (Biorad, San Diego, CA, USA). The membranes were blocked for 1 h at room temperature by 5% fat-free milk and incubated with primary antibodies (ERK1/2, p-ERK1/2, JNK, p-JNK, p38 MAPK, p-p38 MAPK, p65, p-p65, NLRP3, and cleaved caspase-1 were used at 1:1000; RAGE and β-actin were used at 1:3000) overnight at 4 °C accompanied by soft shaking. After washing 3 times per 5 min with PBST, the PVDF membranes were then incubation for 30 min by a HRP-conjugated secondary antibody (1:1000) at room temperature. Finally, immunoreactive bands were visualized with enhanced chemiluminescence (ECL) reagents and detected by ChemiDoc^®^ MP Image Lab (Biorad, San Diego, CA, USA). The image Lab Software (Biorad, San Diego, CA, USA) was used for quantifying band densities.

### 4.9. Statistical Analysis

All data were represented as mean ± SEM from at least three independent experiments. *T*-test or one-way ANOVA test were performed for statistical analysis between groups by Prism 5.0, GraphPad Software (San Diego, CA, USA). A difference considered to be statistically significant was based on *p* value < 0.05.

## Abbreviations

AGEs	advanced glycation end products
AOPP	advanced oxidation protein product
ASC	apoptosis-associated speck-like protein containing CARD
BSA	bovine Serum Albumin
Caspase-1	cysteinyl aspartate specific proteinase 1
C-Caspase-1	cleaved cysteinyl aspartate specific proteinase 1
COX-2	cyclooxygenase 2
DCFH-DA	2’,7’-dichlorodihydrofluorescein diacetate
IL-6	interleukin 6
IL-1β	interleukin 1β
iNOS	inducible nitric oxide synthase
LPS	lipopolysaccharide
MAPK	mitogen activated protein kinase
MB	methylglyoxal-bovine serum albumin
MCP-1	monocyte chemoattractant protein 1
4′MR	4′-methoxyresveratrol; MGO, methylglyoxal
NF-κB	nuclear transcription factor kappa B
NLRP3	nucleotide-binding oligomerization domain (NOD)-like receptor (NLR) pyrin domain containing 3
NO	nitric oxide
NOX1/2	nicotinamide adenine dinucleotide phosphate (NADPH) oxidase 1/2
RAGE	receptor for advanced glycation end products; ROS, reactive oxygen species
TNF-α	tumor necrosis factor alpha

## Appendix A

**Table A1:** Primers used for QPCR analysis.

Gene	Forward Primer	Reverse Primer
**COX2**	CTCACGAAGGAACTCAGCACT	TAGAATCCAGTCCGGGTACAGT
**IL-1β**	GCAACTGTTCCTGAACTCAACT	ATCTTTTGGGGTCCGTCAACT
**IL-6**	AGCCAGAGTCCTTCAGAGAGAT	GCACTAGGTTTGCCGAGTAGAT
**iNOS**	GGCAGCCTGTGAGACCTTTG	GCATTGGAAGTGAAGCGTTTC
**MCP-1**	AGCTCTTTCCTCCACCA	CTACAGCTTCTTTGGGACACCT
**NOX1**	AATGCCCAGGATCGAGGT	GATGGAAGCAAAGGGAGTGA
**NOX2**	CAGGAACCTCACTTTCCATAAGAT	AACGTTGAAGAGATGTGCAATTGT
**RAGE**	AACACAGCCCCCATCCAA	GCTCAACCAACAGCTGAATGC
**TNF-α**	CACCACGCTCTTCTGTCTACTG	CTTTGAGATCCATCGCGTTG
**18S**	GTAACCCGTTGAACCCCATT	CCATCCAATCGGTAGTAGCG
